# On the Bonding Nature in the Crystalline Tri‐Thorium Cluster: Core‐Shell Syngenetic σ‐Aromaticity

**DOI:** 10.1002/anie.202209658

**Published:** 2022-08-03

**Authors:** Xuhui Lin, Yirong Mo

**Affiliations:** ^1^ Sichuan Engineering Research Center for Biomimetic Synthesis of Natural Drugs School of Life Science and Engineering Southwest Jiaotong University Chengdu 610031 China; ^2^ Department of Nanoscience Joint School of Nanoscience and Nanoengineering University of North Carolina at Greensboro Greensboro NC 27401 USA

**Keywords:** Actinide Bonding, Aromaticity, Core–Shell Syngenetic Model, Resonance Energy, Valence Bond

## Abstract

A unique thorium‐thorium bond was observed in the crystalline tri‐thorium cluster [{Th(η^8^‐C_8_H_8_)(μ_3_‐Cl)_2_}_3_{K(THF)_2_}_2_]_∞_, though the claim of σ‐aromaticity for Th_3_ bond has been questioned. Herein, a new type of core–shell syngenetic bonding model is proposed to describe the stability of this tri‐thorium cluster. The model involves a 3c–2e bond in the Th_3_ core and a multicentered (ThCl_2_)_3_ charge‐shift bond with 12 electrons scattering along the outer shell. To differentiate the strengths of the 3c–2e bond and the charge‐shift bond, the block‐localized wavefunction (BLW) method which falls into the ab initio valence bond (VB) theory is employed to construct a strictly core/shell localized state and its contributing covalent resonance structure for the Th_3_ core bond. By comparing with the σ‐aromatic H_3_^+^ and nonaromatic Li_3_^+^, the computed resonance energies and extra cyclic resonance energies confirm that this Th_3_ core bond is truly delocalized and σ‐aromatic.

## Graphical abstract

A core–shell syngenetic bonding model is proposed to describe the chemical bond in a stable crystalline tri‐thorium cluster. It involves a 3c–2e Th_3_ core bond and a multicenter (ThCl_2_)_3_ charge‐shift bond with 12 electrons scattering along the outer shell. The resonance energies and extra cyclic resonance energies from the BLW method further confirm that this novel 3c–2e Th_3_ core bond is delocalized and σ‐aromatic.

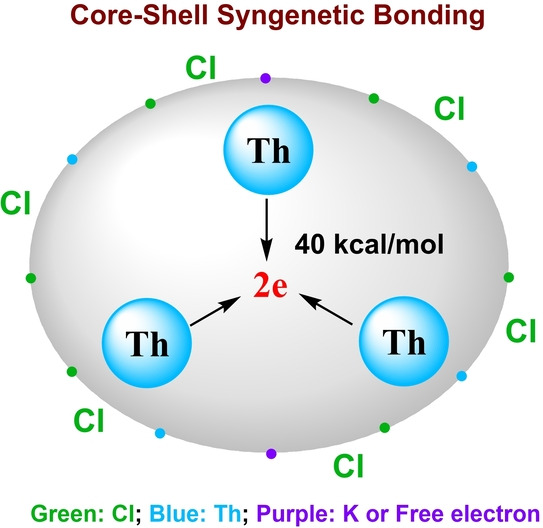

The nature of chemical bonding and its related chemical reactivity is of central interest in chemistry.^[1]^ As the majority in the periodic table, metals form bonds among themselves which have been fascinating chemists for nearly 180 years, as the metal‐containing bonds concern the understanding of variable electronic structures,^[2]^ catalysis,^[3]^ material design^[4]^ and biochemical processes.^[5]^ Different from the well‐established chemical bonding with *d* transition metals, knowledge of actinide‐actinide and actinide‐ligand bonds is still very limited and has been debated for decades.^[6]^ Most recently, the first thorium‐thorium bonding (Th_3_) in the crystalline tri‐thorium cluster [{Th(η^8^‐C_8_H_8_)(μ_3_‐Cl)_2_}_3_{K(THF)_2_}_2_]_∞_ (**3**) was prepared and isolated under mild experimental conditions by Boronski et al., and its remarkable stability was interpreted in terms of the three‐center two electron (3c–2e) σ‐aromatic bond.^[7]^ This bonding mechanism was supported by the theoretical studies of related analogues [{Th(C_8_H_8_)(Cl)_2_}_3_]^2−^ (**3′**) and [{Th(C_8_H_8_)(Cl)_2_}_3_K_2_] (**3′′**). But this unique delocalized Th_3_ bonding model challenges theoretical predictions that actinide‐actinide bond should be very weak and localized,^[8]^ and it still “remains experimentally unproven and computationally questionable”.^[9]^ Notably, Cuyacot and Foroutan‐Nejad argued that **3** is stable but not aromatic,^[10]^ as the negative nucleus‐independent‐chemical shifts (NICS),^[11]^ characteristic for aromaticity, mainly come from the local circulations from the surrounding Th−Cl bonds and the magnitude of the magnetically‐induced paratropic ring current inside the Th_3_ unit is marginal. Moreover, the Raman spectrum for the Th_3_ bond was also observed in [{Th(η^8^‐C_8_H_8_)(μ_3_‐Cl)_2_}_3_K_2_]^2+^ (3*) and [{Th(η^8^‐C_8_H_8_)(μ_3_‐Cl)_2_}_3_Ar_2_] (**3^†^**) without Th−Th bonding. Alternatively, Szczepanik suggested that the chemical bonding in the [Th_3_Cl_6_] cage should be ascribed to a multicenter (ThCl_2_)_3_ charge‐shift bond rather than a σ‐aromatic Th_3_ bond.^[9]^

However, there are two strong proofs supporting the 3c–2e aromatic explanation. One comes from the orbital analysis. The highest occupied molecular orbital (HOMO) in **3′** or **3′′** corresponds to a 3c–2e Th_3_ aromatic bonding motif,^[12]^ but it becomes the lowest unoccupied molecular orbital (LUMO) in [{Th(C_8_H_8_)(Cl)_2_}_3_] (**3^≠^**) or 3* with two electrons removed from **3′** or **3′′** respectively, as shown in Figures 1a. The other is the structural data. The Th−Th bond lengths in **3**, **3′** and **3′′** (3.991 Å, 3.942 Å and 4.035 Å respectively) are much shorter than those in non‐bonded systems 3*, **3^≠^** and **3^†^** (4. 560 Å, 4.392 Å and 4.393 Å respectively), and are only a little longer than twice the Th single‐bond radius of 175 pm^[13]^ but shorter than twice the average covalent atomic radius for Th (206 pm) based on experimental data.^[14]^ It is also interesting to note that a new class of tri‐metallofullerene cation Ln_3_@C_80_^+^ with a 3c–2e lanthanide‐lanthanide bond has been reported very recently.^[15]^

**Figure 1:**
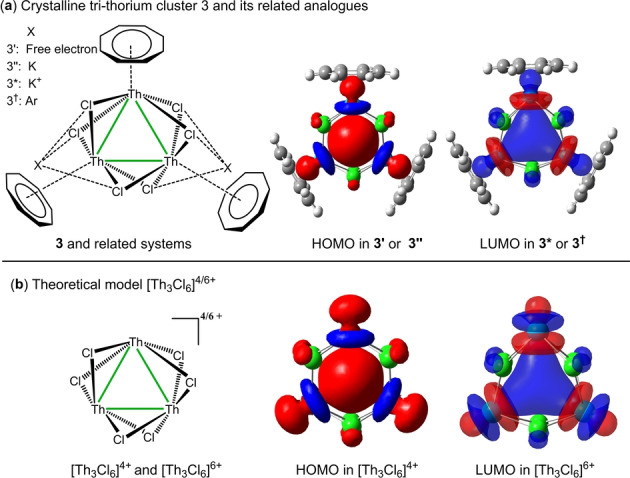
a) Crystalline tri‐thorium cluster [{Th(η^8^‐C_8_H_8_)(μ_3_‐Cl)_2_}_3_{K(THF)_2_}_2_]_∞_ (**3**) and its related analogues; b) theoretical model systems [Th_3_Cl_6_]^4/6+^ used in this work. The HOMOs and LUMOs are plotted at the isovalue of 0.03 a.u.

The controversy in the above centers on the nature of the chemical bonding in the [Th_3_Cl_6_] cage. More precisely, whether this novel Th_3_ cluster is σ‐aromatic or not. The concept of aromaticity has been well recognized in metal clusters^[16]^ particularly in square planar coinage metal cluster^[17]^ and even in [Th@Bi_12_]^4−^ cluster^[18]^ and actinide 2‐metallabiphenylenes compounds,^[19]^ though its acceptance is often accompanied with suspicions. However, it has been found that aromaticity is associated with a range of peculiar magnetic, structural, energetic, and electronic properties,^[20]^ and numerous probes have thus been proposed to establish the concept of aromaticity. Among various criteria, NICS is the most popular and convenient one, though it does not fit for all. Notably, NICS fails to access the aromaticity in small clusters such as Al_2_X_6_ (X=F, Cl, Br, I) cluster, planar (HF)_3_ ring and the crystalline tri‐thorium cluster in this work.[^10^, ^21^] As a consequence, the NICS values cannot be used to examine the 3c–2e Th_3_ bond and justify whether the Th_3_ core is σ‐aromatic or non‐aromatic. Since the concept of aromaticity originates from the unusual molecular stability, ultimately the energetic gain due to the delocalized electrons in closed circuits should be used to affirm the aromaticity. Along this direction, the energy change due to the electron delocalization in Th_3_ is expected to provide a conclusive prediction for the aromaticity in the Th_3_ cluster. Specifically, the extra cyclic resonance energy (ECRE), defined as the RE difference between a cyclic compound and its appropriate acyclic reference, can differentiate aromatic, non‐aromatic and anti‐aromatic compounds based on its sign and magnitude.^[22]^ In this regard, we resort to the ab initio valence bond (VB) theory^[23]^ to derive ECRE and seek an improved understanding of the bonding nature in this unique Th_3_ cluster, as it can construct wave functions for Lewis (resonance or electron‐localized) structures with strictly localized atomic or fragmental orbitals. Notably, the block‐localized wavefunction (BLW) method,[^1c^, ^24^] which is the simplest variant of ab initio VB theory, can define and optimize a particular resonance structure at the DFT level. It should be noted that the current BLW method may not work well if basis functions lose atomic characteristics, e.g., when a complete (infinite) basis on a single center for a molecular system is used. Tests with modest basis sets from 6–31G(d) to 6–311+G(d,p) and cc‐pVTZ showed the basis set dependence is generally trivial, as long as the basis sets are atomic.^[25]^

We firstly investigated the bonding features of the novel 3c–2e Th_3_ bond. The electronic configuration for Th is 6*d* ^2^4 *s* ^2^ with four valence electrons, in which two of them are taken by the ligand C_8_H_8_ so the latter forms an aromatic system with 10 π electrons.^[9]^ The remaining two valence electrons (six in total) would participate in the formation of the focused [Th_3_Cl_6_] cage. Since each electronegative chloride atom need one electron to saturate its valency, all six valence electrons from the three Th ions are grasped by Cl atoms and thus no Th_3_ bonding or 3c–2e HOMO is available in 3* and **3^≠^**. In contrast, there are two electropositive potassium atoms in **3** and **3′′** which can donate two electrons to Cl atoms. In other words, two electrons eventually remain in the Th_3_ cluster, leading to a 3c–2e bond. Similarly, for **3′**, in which the two K atoms are replaced by two free electrons, would also exhibit the 3c–2e Th_3_ bond, which is expected to be a little stronger than that in **3** and **3′′**. This is evidenced by the shortest Th−Th distance in **3′** among them. If the two K atoms in **3′** was replaced by argons (**3^†^**), the optimal Th−Th distance is nearly identical to that in **3^≠^**, because Ar atoms are inert to share electrons with Cl ligands. Thus, the prerequisite for the 3c–2e Th_3_ bond is two extra electrons from additional groups that share with chloride atoms, and the chemical bonding in the [Th_3_Cl_6_] cage is best defined in terms of core–shell syngenetic bonding, including a delocalized 3c–2e bond in the Th_3_ core and a multicenter (ThCl_2_)_3_ charge shift bond with 12 electrons scattering along the outer shell, as shown in Figure 2. In this regard, the tri‐thorium cluster is very similar to a metallofullerene with one metal cluster encapsulated in a fullerene.^[26]^

**Figure 2:**
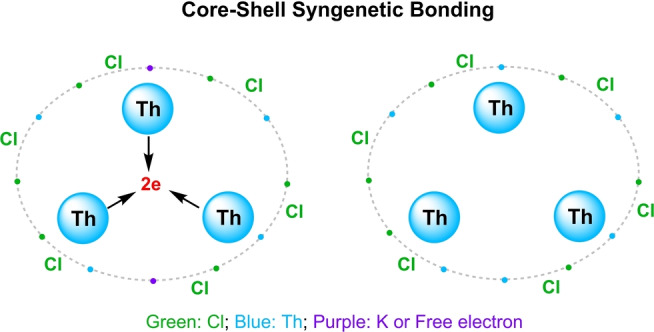
The core–shell syngenetic bonding model, where the dots represent electrons belonging to corresponding atoms.

To validate this bonding model, we simplified system **3** as [Th_3_Cl_6_K_2_]^6+^, where the C_8_H_8_ ligands and two valence electrons of each Th were removed. But this [Th_3_Cl_6_K_2_]^6+^ is unstable and decomposes to [Th_3_Cl_6_]^4+^ and K_2_^2+^ in the process of geometry optimization, and the optimal [Th_3_Cl_6_]^4+^ shares the similar electronic structure and molecular geometry with **3**. In particular, the HOMO in [Th_3_Cl_6_]^4+^ also corresponds to a 3c–2e Th_3_ bond and the Th−Th bond length is reduced to 3.684 Å, indicating enhanced bonding in the Th_3_ core. It is worthy to note that the orbital energy of HOMO for [Th_3_Cl_6_]^4+^ is negative, while it is positive for **3′**, for which Boronski et al. choose **3′′** rather than **3′** as a theoretical model for **3**.^[7]^ As predicted in pretext, if we remove two electrons, the 3c–2e HOMO would degrade to the LUMO in [Th_3_Cl_6_]^6+^ and the Th−Th bond length elongates to 4.310 Å. Thus, models [Th_3_Cl_6_]^4+^ and [Th_3_Cl_6_]^6+^ can be considered as the prototypes of tri‐thorium clusters with and without the 3c–2e Th_3_ bond.

To gain insights into the mechanistic details of the core–shell syngenetic bonding, we followed the evolution of orbitals from deformed but isolated monomers in the complexes without and with the 3c–2e Th_3_ bond, block‐localized monomers in the BLW state, to the final dimer. Figures 3 and 4 showed the “in situ” orbital correlations for [Th_3_Cl_6_]^4+^ and **3′′**. As shown in Figures 3, when three Th^2+^ ions approach to each other, they form an equilateral structure [Th_3_]^6+^ with two degenerate HOMOs and one HOMO‐1 which eventually evolves to be the HOMO in the dimer. From the deformed [Th_3_]^6+^ monomer in the non‐bonded [Th_3_Cl_6_]^6+^ to in the bonded [Th_3_Cl_6_]^4+^, the HOMOs lower their energy levels by ≈2 eV. For the Cl_6_ monomer in the non‐bonded [Th_3_Cl_6_]^6+^, there are three low‐lying unoccupied orbitals tending to accept electrons, and the energy gap between LUMO and degenerate (LUMO+1)s is only 0.2 eV. However, when two electrons (free electrons for [Th_3_Cl_6_]^4+^ or electrons from K atoms for **3′′**) fill the LUMO, the energies of all three orbitals (now one HOMO and two degenerate LUMOs) increase and the energy gap between them expands to 0.5 eV. Similar orbital shifts are observed in **3′′** (Figures 4). Interestingly, when [Th_3_]^6+^ and [Cl_6_]^2−^ or [{Th−C_8_H_8_}_3_]) and [Cl_6_K_2_] are put together, the mutual electrostatic fields with the addition of Pauli repulsion not only further expand the energy gaps notably for the HOMO–LUMO gap in [Cl_6_]^2−^, but also reshuffle the orders of energy levels for [Th_3_]^6+^ or [{Th−C_8_H_8_}_3_], as the HOMO‐1 corresponding to the 3c–2e bonding in Th_3_ is pushed up to become the HOMO. These block‐localized “in situ” frontier orbitals are finally ready to interact (shown in dashed green frames). The two occupied and degenerate (HOMO‐1)s interact with the degenerate LUMOs of [Th_3_]^6+^, confirming electron transfers from the core Th_3_^6+^ or [{Th−C_8_H_8_}_3_] to [Cl_6_]^2−^ or [Cl_6_K_2_], respectively. Due to the electron transfer, [Th_3_Cl_6_]^4+^ or **3′′** can now be viewed as the combination of [Th_3_]^10+^ and [Cl_6_]^6−^ or [{Th−C_8_H_8_}_3_]^4+^ and [Cl_6_K_2_]^4−^, as in the following discussion of σ‐aromaticity. While Figures 3 and 4 show that [Th_3_Cl_6_]^4+^ and **3′′** follow similar orbital evolutions with minor differences in the energy levels, for [Th_3_Cl_6_]^6+^ and 3*, the three HOMOs of the [Th_3_]^6+^ (or [{Th−C_8_H_8_}_3_]) would interact with the three LUMOs of [Cl_6_] (or [Cl_6_K_2_)^2+^), leading the transfer of all six electrons from Th_3_ to the Cl ligands. Consequently, the HOMO‐1 in [Th_3_]^6+^ (or [{Th−C_8_H_8_}_3_]) degrades to the LUMO in final dimers. In other words, there is no 3c–2e bond in [Th_3_Cl_6_]^6+^ or 3*.

**Figure 3:**
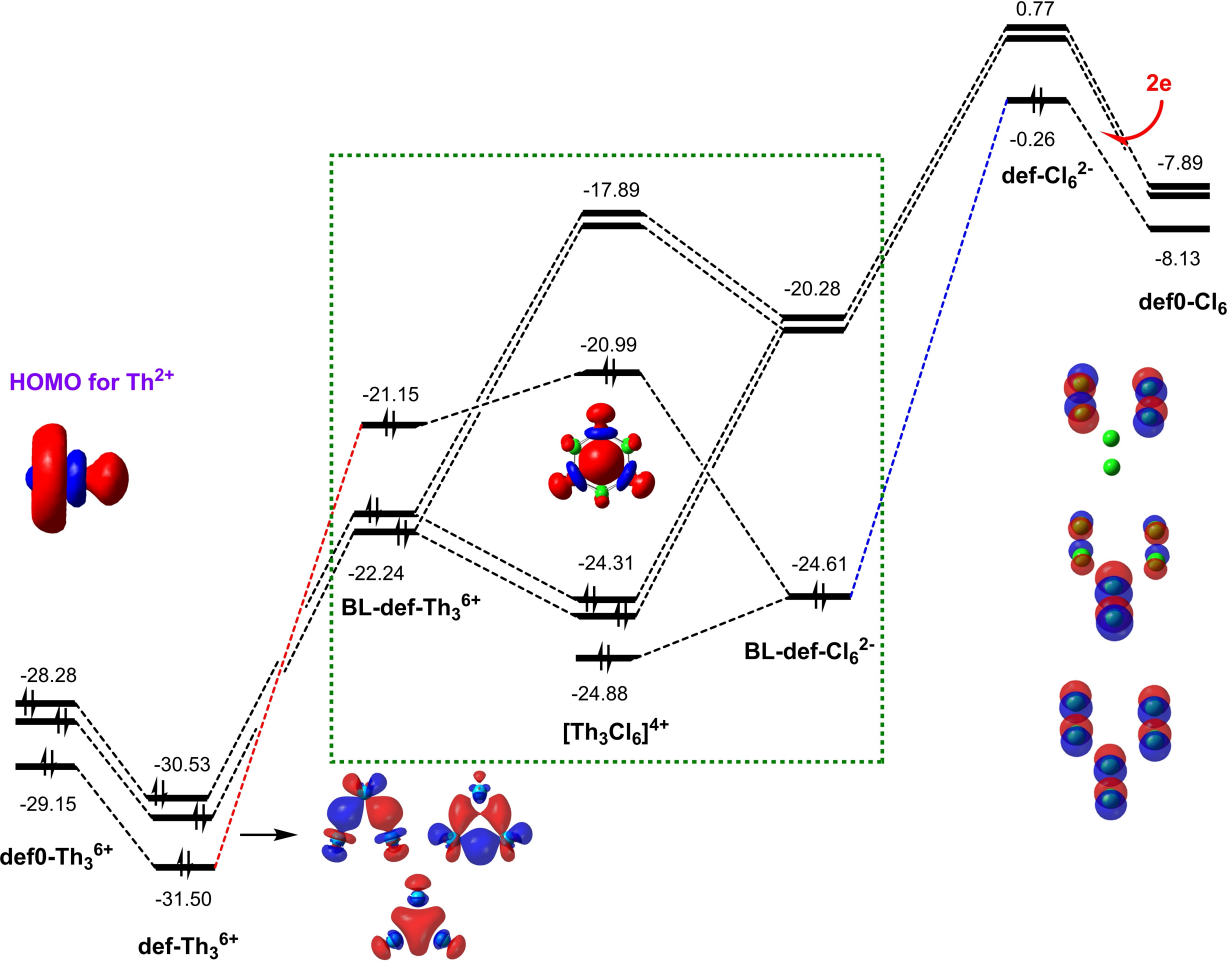
“In situ” orbital correlation diagram for the complex of [Th_3_]^6+^ and [Cl_6_]^2−^, in which def0‐ and def‐ refer to deformed monomers which are isolated from the complexes [Th_3_Cl_6_]^6+^ and [Th_3_Cl_6_]^4+^, respectively, and BL‐refer to block‐localized state of the complex [Th_3_Cl_6_]^4+^. Energies are at eV unit.

**Figure 4:**
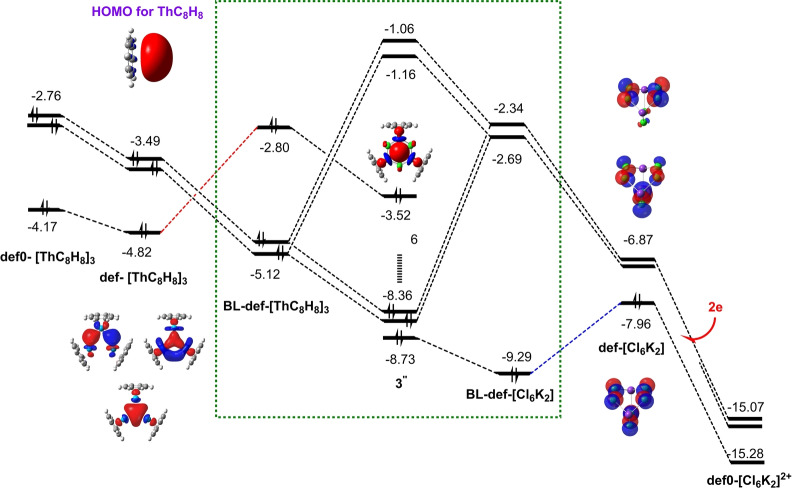
“In situ” orbital correlation diagram for the complex of [{Th−C_8_H_8_}_3_] and [Cl_6_K_2_], def0‐ and def‐ refer to deformed monomers which are isolated from the complexes 3* and **3′′**, respectively, and BL‐ refer to block‐localized state of the complex **3′′**, the number “6′′ in the middle refers to six molecular orbitals for Th−C_8_H_8_ bonds. Energies are at eV unit.

With the confirmation of the existence of Th_3_ core bonding, we employed the BLW method to quantify the σ‐aromatic strength therein. According to the core–shell syngenetic bonding model, we constructed the strictly core/shell localized state, in which the whole system is divided into two blocks. One refers to the shell block i.e., Cl^6−^ (for **3′, 3^≠^**, [Th_3_Cl_6_]^4+^ and [Th_3_Cl_6_]^6+^), [Cl_6_K_2_]^4−^ (for **3′′** and 3*) or [Cl_6_Ar_2_]^6−^ (for **3^†^**) where the valency of all Cl atoms is saturated, while the other involves the remaining core block. In details, the core block corresponds to [Th_3_]^10+^ for [Th_3_Cl_6_]^4+^, [(Th−C_8_H_8_)_3_]^4+^ for **3′** or **3′′**, and [Th_3_]^12+^ or [(Th−C_8_H_8_)_3_]^6+^ for other systems without Th_3_ bonding. Therefore, the energy change (−Δ*E*_Cl→Th_) from this BLW state to the delocalized DFT state results from the electron movement from saturated Cl ligands to the Th_3_ core, which was recognized as the strength for the multicenter [ThCl_2_]_3_ charge shift‐bond by Szczepanik.^[9]^ Since the core block in BLW state only contains one 3c–2e bond in Th_3_ apart from the inner core electrons and Th−C_8_H_8_ ligand bonds, we further built its contributing covalent (BLW^cov^) and ionic (BLW^ion^) resonance structures by localizing the two electrons on two neighbouring Th atoms (or Th−C_8_H_8_) or one particular Th atom (or Th−C_8_H_8_) (Scheme 1). Accordingly, the energy difference between a BLW state and its BLW^cov^ (−Δ*E*_RE_^cov^) or BLW^ion^ (−Δ*E*_RE_^ion^) state at the same DFT geometry is the vertical resonance energy (VRE), which is a probe for the magnitude of electron delocalization.

**Scheme 1:**
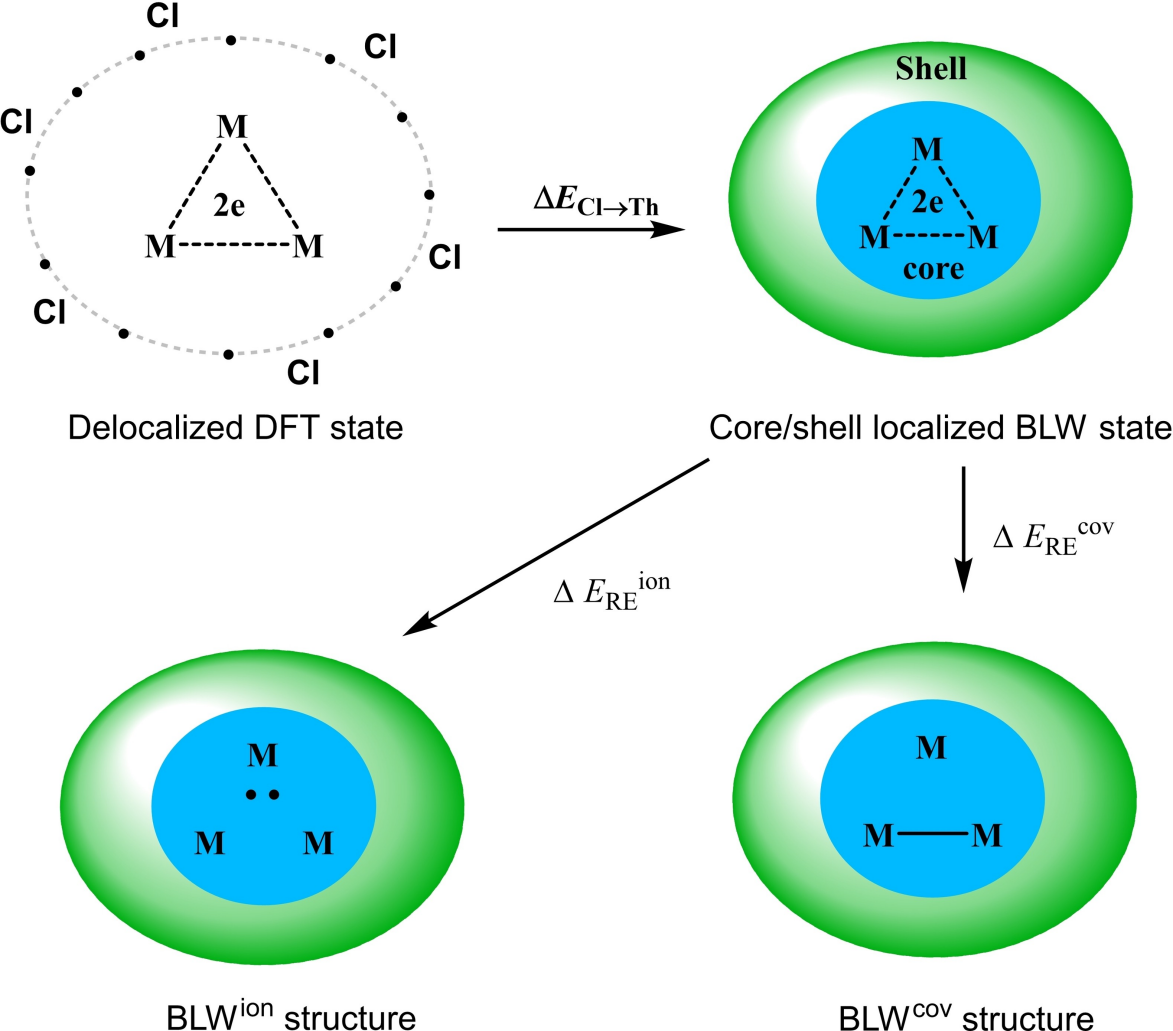
The strictly core‐/shell localized state and its corresponding covalent and ionic resonance structures for the Th_3_ core bond in the BLW computations, where M represents Th atom or Th−C_8_H_8_ group.

Given the prominent Cl→Th electron transfer effect, Szczepanik suggested that the total electron delocalization within [Th_3_Cl_6_] have nothing to do with the σ‐aromatic Th_3_ bond. Indeed, our computations showed that the energetic gains (Δ*E*_Cl→Th_ in Table 1) ranges from 175 to 520 kcal mol^−1^, considering bonding involving 6 pairs of electrons. This electron transfer process can be visualised with electron density difference (EDD) maps between delocalized DFT states and core/shell localized BLW states (see Figure S1 in Supporting Information). But it is premature to defy the electron delocalization within the Th_3_ cluster just because of the overwhelmingly strong Cl→Th electron transfer effect. The vertical resonance energies (VREs) with reference to the covalent structure (Δ*E*_RE_^cov^) for [Th_3_Cl_6_]^4+^, **3′** and **3′′** are 48.7, 42.9 and 40.4 kcal mol^−1^, respectively. The large values of VREs indicate the significant strength of the 3c–2e bond. For the sack of comparison, we also evaluated the VREs for the isolated core blocks [Th_3_]^10+^ in [Th_3_Cl_6_]^4+^ and [(Th−C_8_H_8_)_3_]^4+^ in **3′** and **3′′** without outer shells, whose Δ*E*_RE_^cov^ values are 39.8, 32.5, and 29.8 kcal mol^−1^. The VREs for the isolated core blocks are very similar to those in their corresponding complexes. For comparison, the VREs for the σ‐aromatic H_3_^+^ and non‐aromatic Li_3_^+^ are 85.9 and 13.4 kcal mol^−1^, respectively.

**Table 1:**
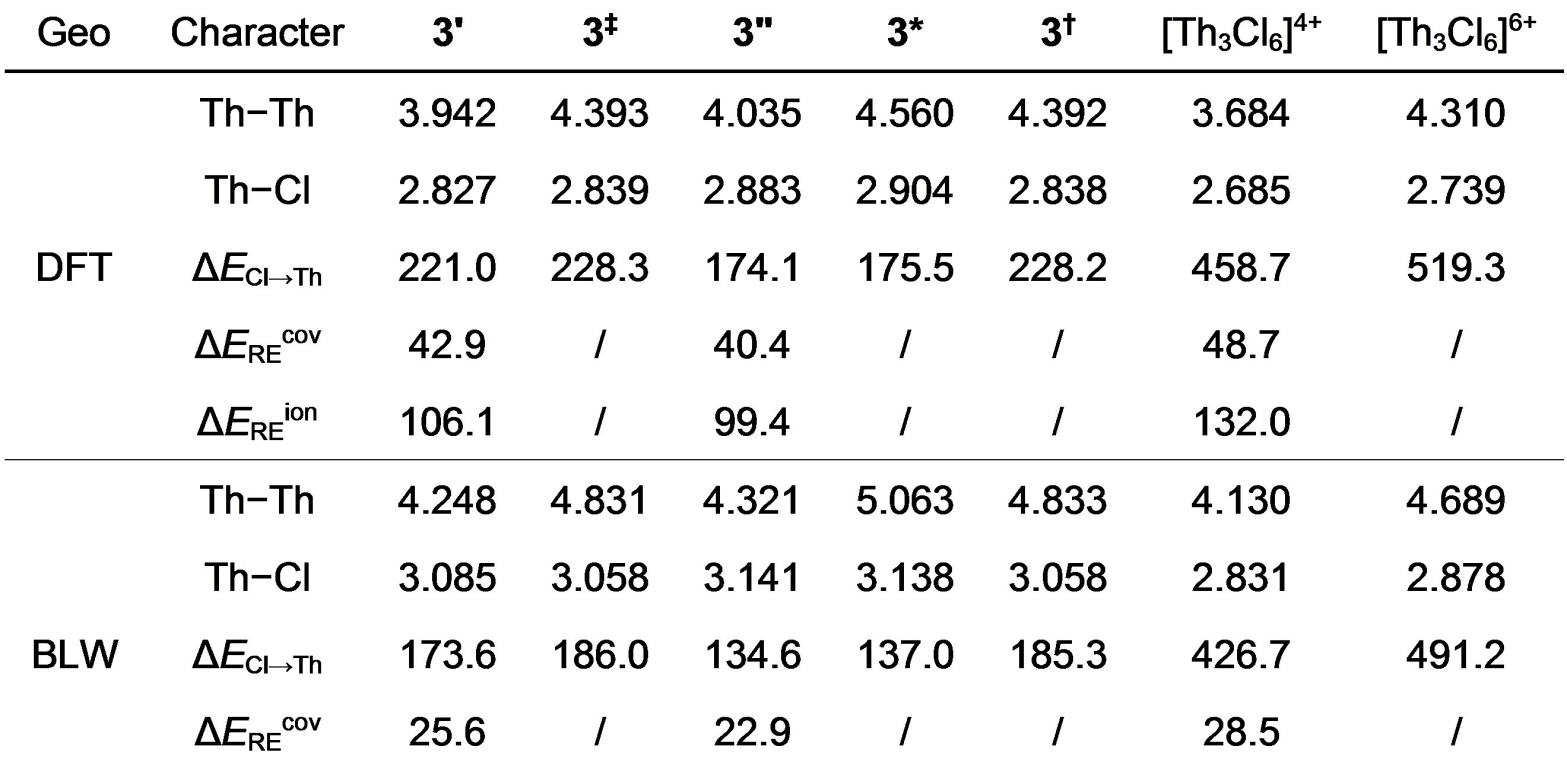
Major bond lengths (in Å) for optimal geometries with regular DFT and BLW methods and corresponding vertical and adiabatic electron delocalization energies (in kcal mol^−1^) at PBE0‐D3(BJ) level.

In order to examine the long‐range exchange and fully relativistic effects, we also calculated the delocalization energy for [Th_3_Cl_6_]^4+^ with the *ω*B97X‐D^[27]^ functional and the standard PBE0 functional plus the Douglas‐Kroll‐Hess 3^rd^‐order (DKH3)^[28]^ correction. Notably, the Δ*E*_Cl→Th_ and Δ*E*_RE_^cov^ with *ω*B97X−D (509.6 and 50.4 kcal mol^−1^) and PBE0 (DKH3) (475.5 and 74.7 kcal mol^−1^) are comparable to and even higher than those with the standard PBE0 functional (458.7 and 48.7 kcal mol^−1^). Thus, the standard PBE0 method can provide reliable and convicing resonance energies, though it may underestimate the delocalization to some extent due to the incomplete consdieration of the relativistic effects.

To further investigate the Cl→Th electron transfer effect on the Th_3_ core bond, we re‐optimized the geometries of the BLW state and its corresponding BLW^cov^ resonance structure, in which the Cl→Th electron transfer is quenched. For the BLW geometries, the Th−Th distances are stretched by about 0.3–0.5 Å, indicating comparable Cl→Th electron transfer effect on the Th_3_ bond for all systems. In particular, the Th−Th distances in [Th_3_Cl_6_]^4+^, **3′** and **3′′** elongate to 4.130 Å, 4.248 Å and 4.321 Å respectively, which are still shorter than those in the systems without the Th_3_ core bond. Moreover, the adiabatic resonance energy (ARE) for Th_3_ core bond (28.5 kcal mol^−1^, 25.6 kcal mol^−1^, 22.9 kcal mol^−1^ for [Th_3_Cl_6_]^4+^, **3′** and **3′′** respectively), deriving from the energy difference between an optimal BLW geometry and its corresponding optimal BLW^cov^ structure, is still very appreciable. In other words, the multicenter [ThCl_2_]_3_ charge shift‐bond cannot purge the electron delocalization in the Th_3_ core, though it indeed influences the strength of the 3c–2e Th_3_ bond.

To visualize the electron delocalization from covalent structure to the delocalized 3c–2e Th_3_ bond, we plotted the EDD maps between the BLW state and its corresponding BLW^cov^ state (Figures 5). It is obvious that the electron density expands from a particular Th−Th bond to the whole Th_3_ unit. Moreover, a shorter Th−Th bond length (3.643 Å, 3.988 Å and 4.026 Å for [Th_3_Cl_6_]^4+^, **3′** and **3′′** respectively) corresponding to the covalent bond and two much longer Th−Th bond lengths 4.395 Å, 4.600 Å and 4.750 Å for [Th_3_Cl_6_]^4+^, **3′** and **3′′** respectively) are observed in the optimal covalent structures. The optimal covalent structures are well‐consistent with our chemical intuition about what a 3c–2e covalent bond should be.

**Figure 5:**
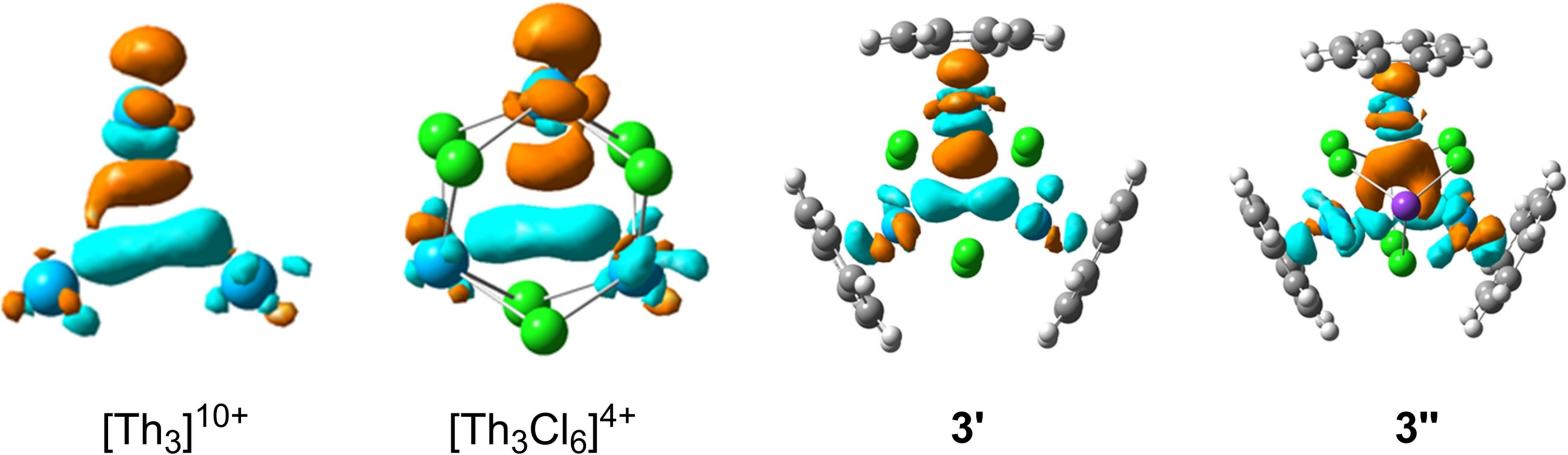
EDD maps with an isovalue of 0.005 a.u. for [Th_3_]^10+^ and [Th_3_Cl_6_]^4+^ and 0.004 a.u. for **3′** and **3′′** showing the movement of electron density (orange for gain and cyan for loss) from a particular Th−Th single bond to the whole Th_3_ unit.

However, the authentication of electron delocalization in the Th_3_ core cannot be simply used as evidence for the existence of σ‐aromaticity, as similar electron delocalization may also exhibit in corresponding acyclic analogues. In the original paper by Boronski et al.,^[7]^ the authors compared the calculated NICS(0) values of **3′′** (−15.23 ppm)^[7]^ and σ‐aromatic H_3_^+^ (−33.38 ppm)^[11b]^ and Li_3_^+^ (−11.1 ppm).^[11b]^ H_3_^+^ has been widely recognized as a σ‐aromatic paradigm, but Li_3_^+^ has been claimed as a nonaromatic system despite its negative NICS.^[29]^ Since NICS fails to access the σ‐aromaticity in this three‐membered metal cluster, extra cyclic resonance energy (ECRE), defined as the RE difference between a cyclic compound and its appropriate acyclic reference, is more suitable for assessing the σ‐aromaticity (Figures 6a). Specifically, a positive ECRE represents the magnitude of aromaticity, whereas negative ECRE corresponds to an antiaromatic system. The ECRE for a nonaromatic system thus should be around zero.

**Figure 6:**
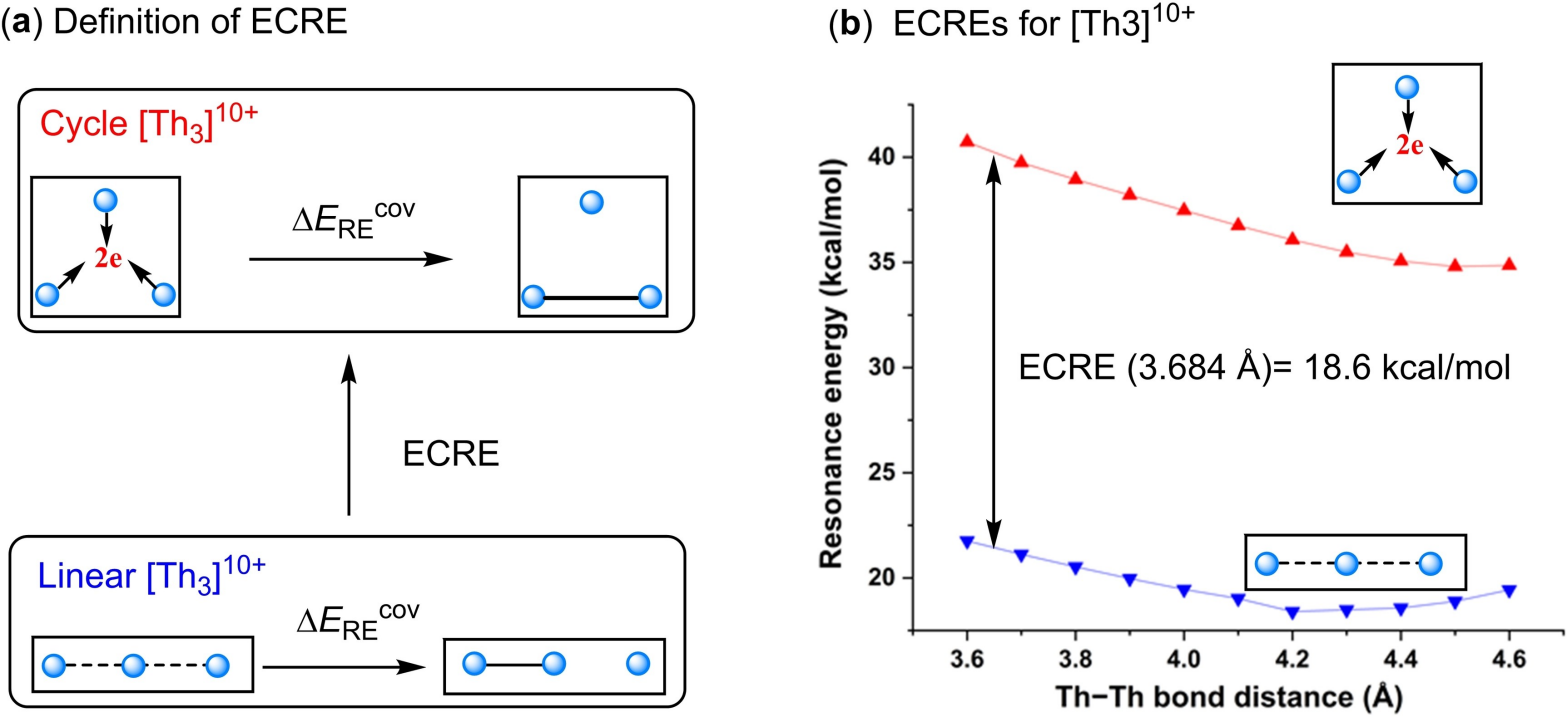
The evaluation of the extra cyclic resonance energy (ECRE) for [Th_3_]^10+^.

We evaluated the ECREs for the core [Th_3_]^10+^ together with the σ‐aromatic H_3_^+^ and Li_3_^+^, where linear X−X−X (X=Th, H or Li) systems are considered as their acyclic references with the same bond lengths as in cyclic analogues. Our computed ECREs for H_3_^+^ and Li_3_^+^ are 31.9 kcal mol^−1^ and 0.2 kcal mol^−1^, respectively. These data are consistent with the general view that H_3_^+^ is σ‐aromatic yet Li_3_^+^ is nonaromatic. Since the Th_3_ core [Th_3_]^10+^ carries many positive charges and it is not possible to obtain a stable cluster, we calculated the resonance energy for the cyclic [Th_3_]^10+^ and its corresponding linear [Th−Th−Th]^10+^ structure at variable Th−Th distances from 3.6 Å to 4.6 Å. Results are shown in Figures 6b. It is obvious that the RE for the cyclic structure is much higher than the value for the linear structure of [Th_3_]^10+^, confirming the σ‐aromaticity for the Th_3_ core bond. Specifically, the ECRE for [Th_3_]^10+^ at the Th−Th bond length (3.684 Å) in [Th_3_Cl_6_]^4+^ is 18.6 kcal mol^−1^, which is about 60 % of the σ‐aromaticity in H_3_^+^ (31.9 kcal mol^−1^). It should be noted that another reasonable acyclic system is linear [Th−Th−Th−Th]^14+^ with two electrons delocalizing among three Th−Th bonds as that in cyclic [Th_3_]^10+^, and the corresponding ECRE increases to 28.8 kcal mol^−1^, further indicating the σ‐aromaticity in Th_3_ core bond.

In summary, the presence of σ‐aromaticity for the Th_3_ bond in a crystalline tri‐thorium cluster prepared by Boronski et al.^[7]^ was extensively studied, Foroutan‐Nejad^[10]^ and Szczepanik^[9]^ questioned this claim due to the conflicting arguments by NICS value and Raman spectrum. We generated “in situ” orbital correlation diagrams to unravel the nature of the chemical bonding in the tri‐thorium cluster and proposed a core–shell syngenetic model. The σ‐aromaticity in the Th_3_ core bond was further explored with the BLW method by constructing a strictly core/shell localized state and the contributing covalent resonance structure for the Th_3_ bond. Computational results showed that this 3c–2e Th_3_ bond is truly delocalized as its covalent resonance energy was calculated in the range of 40–50 kcal mol^−1^, lying between the σ‐aromatic extreme H_3_^+^ (85.8) and the nonaromatic Li_3_^+^ (13.3 kcal mol^−1^). Notably, the extra cyclic resonance energies for Th_3_^10+^, H_3_^+^ and Li_3_^+^ are 18.6 kcal mol^−1^, 31.9 kcal mol^−1^ and 0.2 kcal mol^−1^, respectively, confirming the considerable σ‐aromaticity in the Th_3_ core bonding.

## Conflict of interest

The authors declare no conflict of interest.

## Supporting information

As a service to our authors and readers, this journal provides supporting information supplied by the authors. Such materials are peer reviewed and may be re‐organized for online delivery, but are not copy‐edited or typeset. Technical support issues arising from supporting information (other than missing files) should be addressed to the authors.

Supporting Information

## Data Availability

The data that support the findings of this study are available in the Supporting Information of this article.
