# Supplementary material for: On the Bonding Nature in the Crystalline Tri‐Thorium Cluster: Core‐Shell Syngenetic σ‐Aromaticity
Source: Angew Chem Int Ed Engl. 2022 Aug 3;61(37):e202209658. doi: 10.1002/anie.202209658 (PMC9541753; doi:10.1002/anie.202209658)
Supplement: Supplementary file 1 — Supporting Information [file ANIE-61-0-s001.pdf]

Angewandte Chemie
Eine Zeitschrift der Gesellschaft Deutscher Chemiker

Supporting Information

On the Bonding Nature in the Crystalline Tri-Thorium Cluster: Core-Shell Syngenetic σ-Aromaticity

X. Lin, Y. Mo**

Methodology

Block-localized wavefunction (BLW) method. Molecular orbital (MO) theory assumes that all electrons are in MOs which are delocalized to the whole system, whereas valance bond (VB) theory starts from localized atomic orbitals to build Lewis (resonance) structures and describes a conjugated system with several resonance structures.[1] Each resonance structure can be defined with a Heitler-London-Slater-Pauling (HLSP) function which is essentially a combination of a number of Slater determinants. To simplify the computational costs and combine the advantages of both MO and VB theories, we proposed the BLW method where a BLW corresponds to a unique electron-localized diabatic state (usually the most stable resonance state).[2] The fundamental assumption is that all electrons and primitive basis functions (χ) can be divided into k subgroups (blocks), and each MO is block-localized and expanded in only one block. Assuming that there are m_i basis and n_i electrons for block i, we can express block-localized MOs for this block as

$$\varphi_j^i = \sum_{\mu=1}^{m_i} C_{j\mu}^i \chi_\mu^i \tag{1}$$

Subsequently, the BLW for a closed-shell is defined using a Slater determinant as

$$\Psi^{\mathrm{BLW}} = \det\left|\left(\phi_1^1\right)^2\left(\phi_2^1\right)^2\cdots\left(\phi_{n_1/2}^1\right)^2\cdots\left(\phi_1^i\right)^2\cdots\left(\phi_{n_i/2}^i\right)^2\cdots\left(\phi_{/n_k/2}^k\right)^2\right| = \hat{A}\left[\Phi_1\Phi_2\cdots\Phi_k\right] \tag{2}$$

Orbitals in the same subspace are subject to the orthogonality constraint, but orbitals belonging to different subspaces are nonorthogonal. The BLW method is available at the DFT level with the geometry optimization and frequency computation capabilities.

Computational Details

All geometry optimizations and the harmonic vibrational frequency calculations were performed with Gaussian G16[3] at PBE0 level[4] augmented by Grimme's D3 dispersion corrections[5] with the Becke-Johnson (BJ) damping[6]. The small-core Stuttgart-Bonn relativistic effective-core potential (replacing 60 core electrons) and the associated segmented valence basis sets was adopted for thorium,[7] while the remaining atoms including hydrogen, lithium, carbon, chlorine, potassium, and argon were represented by Def2-TZVP basis set. SuperFine grid and tight

convergence criteria were used in all the self-consistent field (SCF) calculations. All wavefunctions were confirmed to be ground-state minima with stable closed-shell configurations by the procedure implemented in Gaussian (the keyword "Stable"). All BLW computations were employed with the GAMESS software with identical SuperFine grid.[8] In particular, in order to reduce the computational costs, the Def2-TZVP basis set for carbon and hydrogen atoms was replaced by smaller Def2-SVP basis sets for **3** and related analogues in BLW calculations. In order to address the long-range exchange effect and fully relativistic effects, we have also performed the calculations for $[Th_3Cl_6]^{4+}$ at wB97XD[9] level and Douglas-Kroll-Hess 3rd-order (DKH3)[10] with the fully relativistic polarized triple zeta basis[11] respectively.

References

[1] [a] D. Cooper, *Valence bond theory*, Elsevier, **2002**; [b] S. S. Shaik, P. C. Hiberty, *A chemist's guide to valence bond theory*, John Wiley & Sons, **2007**; [c] W. Wu, P. Su, S. Shaik, P. C. Hiberty, *Chem. Rev.* **2011**, *111*, 7557-7593.

[2] [a] Y. Mo, S. D. Peyerimhoff, *J. Chem. Phys.* **1998**, *109*, 1687-1697; [b] Y. Mo, L. Song, Y. Lin, *J. Phys. Chem. A* **2007**, *111*, 8291-8301.

[3] M. J. Frisch, et al., Wallingford, CT, **2016**.

[4] [a] C. Adamo, V. Barone, *J. Chem. Phys.* **1999**, *110*, 6158-6170; [b] J. P. Perdew, K. Burke, M. Ernzerhof, *Phys. Rev. Lett.* **1996**, *77*, 3865-3868.

[5] S. Grimme, J. Antony, S. Ehrlich, H. Krieg, *J. Chem. Phys.* **2010**, *132*, 154104.

[6] [a] S. Grimme, S. Ehrlich, L. Goerigk, *J. Comput. Chem.* **2011**, *32*, 1456-1465; [b] D. G. A. Smith, L. A. Burns, K. Patkowski, C. D. Sherrill, *J. Phys. Chem. Lett* **2016**, *7*, 2197-2203.

[7] [a] W. Küchle, M. Dolg, H. Stoll, H. Preuss, *J. Chem. Phys.* **1994**, *100*, 7535-7542; [b] X. Cao, M. Dolg, H. Stoll, *J. Chem. Phys.* **2003**, *118*, 487-496; [c] X. Cao, M. Dolg, *Journal of Molecular Structure: THEOCHEM* **2004**, *673*, 203-209.

[8] G. M. J. Barca, et al., *J. Chem. Phys.* **2020**, *152*, 154102.

[9] [a] J.-D. Chai, M. Head-Gordon, *J. Chem. Phys.* **2008**, *128*, 084106; [b] J.-D. Chai, M. Head-Gordon, *Phys. Chem. Chem. Phys.* **2008**, *10*, 6615-6620.

[10] [a] M. Douglas, N. M. Kroll, *Ann. Phys.* **1974**, *82*, 89-155; [b] B. A. Hess, *Phys. Rev. A* **1985**, *32*, 756-763; [c] B. A. Hess, *Phys. Rev. A* **1986**, *33*, 3742-3748.

[11] A. Z. de Oliveira, C. T. Campos, F. E. Jorge, I. B. Ferreira, P. A. Fantin, *Comput. Theor. Chem.* **2018**, *1135*, 28-33.

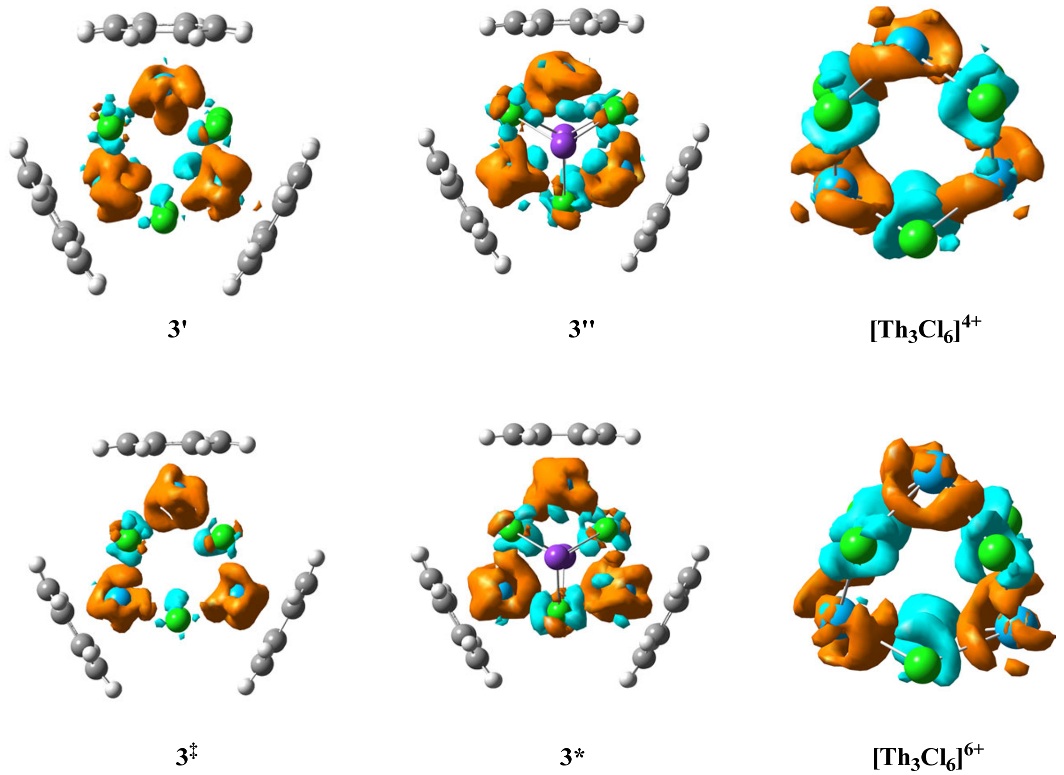

Figure S1. EDD maps with an isovalue of 0.005 a.u. for **3′** and **3**‡, 0.003 a.u. for **3″** and **3*** and 0.008 a.u. for $[Th_3Cl_6]^{4+}$ and $[Th_3Cl_6]^{6+}$ showing the movement of electron density from Cl ligands to Th metal centers.

The optimal geometries and absolute energies at PBE0-D3 level

3′ (DFT) *E* = -3796.0092926501 a.u.

Th	-2.25324600	-0.32094800	-0.00025200
C	-4.19705200	-1.29701900	1.69826900
H	-4.00126500	-1.68001700	2.69550600
C	-4.06175400	-2.28512100	0.71278200
H	-3.78662600	-3.24902900	1.13026700
C	-4.06068400	-2.29279300	-0.69334900
H	-3.78528000	-3.26132300	-1.09990900
C	-4.19506600	-1.31591000	-1.69010800
H	-3.99740700	-1.71030600	-2.68254200
C	-4.39209700	0.07702600	-1.69822100
H	-4.31013100	0.49953900	-2.69538700
C	-4.53654600	1.06392000	-0.71279200
H	-4.53996700	2.06623900	-1.13051700
C	-4.53768800	1.07166000	0.69352800
H	-4.54216500	2.07851100	1.10022100
C	-4.39485800	0.09583500	1.69012100
H	-4.31444800	0.52964700	2.68255100
Th	0.84869300	2.11214100	-0.00000800
C	2.26345300	3.76462500	-1.69758300
H	2.58842900	3.48225800	-2.69470000
C	0.05251800	4.66033200	0.71294100
H	-0.91982900	4.90423500	1.13027300
C	0.04551400	4.66325200	-0.69317400
H	-0.93085100	4.90924600	-1.09986000
C	0.95879500	4.29092600	-1.68974800
H	0.51863800	4.31712700	-2.68228300
C	0.97553300	4.28288200	1.69855300
H	0.54571000	4.30488400	2.69568800
C	3.18995900	3.39559200	-0.71203300
H	4.05951100	2.89699900	-1.12969700
C	3.19687600	3.39227200	0.69428000
H	4.07067900	2.89211300	1.10107600
C	2.28036000	3.75693400	1.69071000
H	2.61538700	3.47003000	2.68319400
Th	1.40454500	-1.79107500	0.00004800
C	2.12921000	-3.84203800	-1.69801200

H	1.72236000	-3.98280600	-2.69509000
C	1.34738300	-4.46075600	-0.71225000
H	0.48101300	-4.96519700	-1.12945800
C	1.34149200	-4.46520700	0.69383600
H	0.47183900	-4.97257300	1.10062900
C	2.11530500	-3.85358500	1.69029600
H	1.69961500	-4.00079200	2.68280700
C	3.22233100	-2.98549100	1.69801900
H	3.45606300	-2.62382800	2.69508600
C	4.00992000	-2.37386600	0.71232300
H	4.70689100	-1.65340000	1.12983100
C	4.01575600	-2.36921300	-0.69404100
H	4.71643700	-1.64632100	-1.10102900
C	3.23661100	-2.97436400	-1.69035600
H	3.47889700	-2.60610500	-2.68295900
Cl	-0.79189500	-1.96953600	1.77084000
Cl	-1.31177400	1.67062400	1.77001900
Cl	-1.31112100	1.66984100	-1.77073500
Cl	-0.79173800	-1.96991400	-1.77093800
Cl	2.10255800	0.29938500	-1.77017800
Cl	2.10194800	0.29906600	1.77048900

3′ (BLW) *E* = -3795.7325894669 a.u.

TH	-2.4283101216	-0.3403874183	-0.0000275772
C	-4.2635284715	-1.3068223197	1.7125459126
H	-4.0304730248	-1.6821689784	2.7131983369
C	-4.1296471482	-2.3047295897	0.7265352030
H	-3.8185306416	-3.2626718582	1.1521523175
C	-4.1270877387	-2.3207260849	-0.6868878855
H	-3.8152685925	-3.2883163556	-1.0897246910
C	-4.2587149877	-1.3458372764	-1.6959552974
H	-4.0222254336	-1.7438187437	-2.6869167290
C	-4.4632659496	0.0535546040	-1.7125619872
H	-4.3484273446	0.4797645313	-2.7134019883
C	-4.6197566953	1.0481366531	-0.7263604053
H	-4.5971108060	2.0552490307	-1.1515814145
C	-4.6217309497	1.0641550691	0.6874639282
H	-4.6012237780	2.0806737741	1.0899355491

C	-4.4692994749	0.0924064329	1.6967658444
H	-4.3577249280	0.5412596523	2.6879190096
TH	0.9174069642	2.2790756120	0.0030173776
C	2.3006140900	3.8332019567	-1.7012590750
H	2.6187763537	3.5169362687	-2.6988539925
C	0.0765535900	4.7383667773	0.7181944177
H	-0.9108456541	4.9542015560	1.1354470463
C	0.0725167570	4.7414824530	-0.6955354586
H	-0.9171678485	4.9593691827	-1.1062887373
C	0.9886867998	4.3616667072	-1.6967994898
H	0.5337566661	4.3562587572	-2.6914802245
C	0.9980275777	4.3532197016	1.7125311489
H	0.5485303390	4.3431312062	2.7096841007
C	3.2310299960	3.4697634919	-0.7071566767
H	4.0925766648	2.9416958680	-1.1248742631
C	3.2347814141	3.4660313598	0.7064188244
H	4.0988302058	2.9363189205	1.1168226457
C	2.3099555589	3.8247132510	1.7074313117
H	2.6333873724	3.5033239833	2.7016576310
TH	1.5134611805	-1.9252089481	-0.0026908737
C	2.1588677186	-3.8995536842	-1.7124835863
H	1.7239488788	-4.0130592737	-2.7096570044
C	1.3784080831	-4.5226233830	-0.7183231423
H	0.4881740697	-5.0010132683	-1.1357794429
C	1.3751920126	-4.5269226743	0.6953013840
H	0.4832472117	-5.0080970906	1.1058772318
C	2.1517257370	-3.9106479006	1.6967680223
H	1.7125375128	-4.0305046016	2.6912861376
C	3.2676908116	-3.0417418907	1.7017573939
H	3.4867430480	-2.6507608338	2.6995545952
C	4.0626815400	-2.4364676847	0.7077981756
H	4.7463072888	-1.6923967942	1.1256986913
C	4.0654805739	-2.4317830718	-0.7059400843
H	4.7510852956	-1.6852930624	-1.1162211954
C	3.2749564887	-3.0308153762	-1.7071999557
H	3.4980545465	-2.6332229837	-2.7014693395
CL	-0.8951538850	-2.2248627027	1.9036177426
CL	-1.4883597468	1.9007701153	1.9003825638
CL	-1.4870133121	1.9030618583	-1.8973369035

CL	-0.8968785951	-2.2228133649	-1.9065566916
CL	2.3900101764	0.3445757410	-1.9004429609
CL	2.3904836042	0.3395106993	1.9009785306

3′ (BLW^{cov})

3″ (DFT) *E* = -4995.5699386954 a.u.

Th	-1.79422000	-1.48519200	0.00000200
C	-3.78335600	-2.22102800	1.69332100
H	-3.96581000	-1.83406100	2.69100900
C	-4.41505700	-1.45571700	0.70364300
H	-4.96115700	-0.61422700	1.11666300
C	-4.41520000	-1.45503000	-0.70259700
H	-4.96128000	-0.61308400	-1.11469600
C	-3.78369600	-2.21942300	-1.69317500
H	-3.96639000	-1.83149300	-2.69043700
C	-2.88574900	-3.30315700	-1.69377700
H	-2.53905200	-3.55400400	-2.69140700
C	-2.25143400	-4.06612000	-0.70406500
H	-1.52569600	-4.75872300	-1.11702100
C	-2.25120500	-4.06663000	0.70206700
H	-1.52534200	-4.75958600	1.11421700
C	-2.88522300	-3.30453900	1.69264000
H	-2.53820000	-3.55629500	2.68993800
Th	2.18330600	-0.81172600	0.00014300
C	4.30338600	-0.84841300	-1.69339800
H	4.34744100	-0.42278000	-2.69103800
C	3.46743700	-3.09646000	0.70382800
H	3.01142000	-3.99001400	1.11676400
C	3.46711400	-3.09692400	-0.70238100
H	3.01078300	-3.99066900	-1.11454000
C	3.81371000	-2.16788200	-1.69286700
H	3.56912000	-2.51998800	-2.69016000
C	3.81436600	-2.16676100	1.69352400
H	3.57021600	-2.51821600	2.69116100
C	4.64666900	0.08251600	-0.70367400
H	4.88362200	1.05732600	-1.11662000

C	4.64664300	0.08301100	0.70245900
H	4.88365500	1.05813100	1.11464500
C	4.30362300	-0.84719000	1.69296800
H	4.34792200	-0.42080800	2.69028700
Th	-0.38949200	2.29689300	-0.00009500
C	-1.41557500	4.15281300	-1.69355400
H	-1.80637800	3.97880100	-2.69125400
C	-2.39378400	3.98561800	-0.70391600
H	-3.35681900	3.70478800	-1.11696400
C	-2.39441000	3.98525600	0.70232400
H	-3.35776600	3.70410400	1.11438600
C	-1.41706200	4.15197400	1.69296000
H	-1.80878700	3.97746500	2.69020100
C	-0.02934300	4.38656600	1.69364200
H	0.39698300	4.35020700	2.69129500
C	0.94932400	4.55027800	0.70397200
H	1.95121700	4.60099100	1.11697100
C	0.94990400	4.55045800	-0.70216600
H	1.95216900	4.60130400	-1.11425400
C	-0.02785700	4.38712600	-1.69279500
H	0.39944300	4.35115900	-2.69005600
Cl	-2.03320800	0.75489500	1.79725200
Cl	0.36254700	-2.13764600	1.79804000
Cl	0.36235100	-2.13757400	-1.79805300
Cl	-2.03298200	0.75524500	-1.79735200
Cl	1.66991000	1.38222200	-1.79797500
Cl	1.66952800	1.38246200	1.79814200
K	-0.00045400	0.00022800	-3.82898800
K	-0.00073400	0.00012000	3.82907500

3″ (BLW)

3″ (BLW^{cov})

$[Th_3Cl_6]^{4+}$ (DFT) *E* = -3982.1436206935 a.u.

Th	0.00000000	1.84227113	-1.06360407
Th	-0.00000000	-1.84227113	-1.06360407
Th	0.00000000	0.00000000	2.12713815

Cl	1.67773312	0.00000000	-2.06410415
Cl	-1.67773312	0.00000000	-2.06410415
Cl	-1.67773312	-1.78766413	1.03214507
Cl	1.67773312	-1.78766413	1.03214507
Cl	-1.67773312	1.78766413	1.03214507
Cl	1.67773312	1.78766413	1.03214507

$[Th_3Cl_6]^{4+}$ (BLW) *E* = -3981.4636222142 a.u.

TH	-0.0000052962	2.0622063750	-1.1927926899
TH	0.0000190356	-2.0623959377	-1.1929889608
TH	-0.0000147852	0.0000037171	2.3904286169
CL	1.6866848189	-0.0000808583	-2.1489975067
CL	-1.6866858162	-0.0000969781	-2.1489745096
CL	-1.6852675431	-1.8552512750	1.0733153293
CL	1.6852670033	-1.8552434909	1.0733361936
CL	-1.6852918391	1.8554543331	1.0735004242
CL	1.6852944218	1.8554041147	1.0734750931

$[Th_3Cl_6]^{4+}$ (BLW^{cov}) *E* = -3981.4181923242 a.u.

TH	0.0000013073	2.3354782536	-1.3478660013
TH	-0.0000040076	-2.0381026584	-0.9249372484
TH	0.0000018029	-0.2179375766	2.2307424020
CL	1.6132540910	0.2113671010	-2.0467387878
CL	-1.6132528718	0.2113716470	-2.0467455078
CL	-1.7939296185	-2.1253296008	1.2282375316
CL	1.7939304302	-2.1253275579	1.2282305929
CL	-1.6136505158	1.8742368160	0.8396879263
CL	1.6136493849	1.8742435759	0.8396910825

$3^{‡}$ (DFT) *E* = -3795.9792621806 a.u.

TH	-2.48388918	-0.51406604	0.00010700
C	-4.25315031	-1.59555712	1.69818812
H	-4.04431229	-1.97172414	2.69344219
C	-4.05801129	-2.56897118	0.70675705
H	-3.73210527	-3.51702725	1.11961208
C	-4.05748529	-2.57161219	-0.69983405

H	-3.73123127	-3.52119925	-1.10888908
C	-4.25193331	-1.60191811	-1.69504612
H	-4.04231329	-1.98174214	-2.68873719
C	-4.53731233	-0.22405002	-1.69768412
H	-4.49509932	0.20410801	-2.69294619
C	-4.74478334	0.74681506	-0.70625405
H	-4.82217235	1.74632512	-1.11912908
C	-4.74522034	0.74944905	0.70032205
H	-4.82287034	1.75051513	1.10938608
C	-4.53844333	-0.21767402	1.69553412
H	-4.49684732	0.21417702	2.68921619
TH	0.79669306	2.40812617	0.00004000
C	2.07486315	4.04112329	-1.69779312
H	2.42448617	3.79038327	-2.69305219
C	-0.19533601	4.79900735	0.70666805
H	-1.17927708	4.99103836	1.11953608
C	-0.19792701	4.79983734	-0.69992305
H	-1.18338409	4.99233436	-1.10895008
C	0.73898905	4.48317232	-1.69515012
H	0.30522902	4.49164732	-2.68883519
C	0.74518005	4.48109732	1.69808812
H	0.31500002	4.48843532	2.69334519
C	3.01933922	3.73517627	-0.70637305
H	3.92353628	3.30224724	-1.11925808
C	3.02186721	3.73422927	0.70019805
H	3.92755528	3.30075024	1.10924208
C	2.08101015	4.03892329	1.69542412
H	2.43415617	3.78689027	2.68910119
TH	1.68714112	-1.89399914	-0.00023800
C	2.46248218	-3.81727027	-1.69817512
H	2.07059815	-3.99472629	-2.69345719
C	1.72525812	-4.48227732	-0.70681105
H	0.89830507	-5.04894736	-1.11974108
C	1.72310112	-4.48405332	0.69976605
H	0.89486407	-5.05172637	1.10873808
C	2.45737218	-3.82161527	1.69504012
H	2.06247615	-4.00148229	2.68868819
C	3.50816125	-2.88577521	1.69776912
H	3.72950527	-2.51689818	2.69304919

C	4.25375130	-2.23014616	0.70640505
H	4.91190435	-1.47397111	1.11933308
C	4.25576931	-2.22826016	-0.70017405
H	4.91515535	-1.47101011	-1.10916908
C	3.51316325	-2.88131621	-1.69545712
H	3.73739727	-2.50988518	-2.68913019
CL	-0.62672505	-1.89405414	1.64501212
CL	-1.32731309	1.49002211	1.64508412
CL	-1.32726010	1.48968411	-1.64498412
CL	-0.62681404	-1.89424714	-1.64508512
CL	1.95409314	0.40462503	-1.64515912
CL	1.95402414	0.40431703	1.64500612

3‡ (BLW) *E* = -3795.6828924744 a.u.

TH	-2.7349622056	-0.5662131244	-0.0001790565
C	-4.4197227307	-1.6355933188	1.7063095545
H	-4.1782852573	-2.0087062835	2.7050317490
C	-4.2269166682	-2.6141902766	0.7081009315
H	-3.8716119340	-3.5607409269	1.1229737495
C	-4.2264367374	-2.6151473541	-0.7065919572
H	-3.8708512879	-3.5622808509	-1.1199310951
C	-4.4186446651	-1.6379394265	-1.7062685537
H	-4.1767407849	-2.0124396457	-2.7043393638
C	-4.7049845594	-0.2517292065	-1.7072841124
H	-4.6303932661	0.1865825828	-2.7059432303
C	-4.9143385359	0.7235826924	-0.7091568977
H	-4.9618785393	1.7336186101	-1.1238885782
C	-4.9146883241	0.7245714246	0.7057097978
H	-4.9624606980	1.7352007594	1.1190040432
C	-4.7059908382	-0.2493674216	1.7053119963
H	-4.6321670587	0.1902695318	2.7034141406
TH	0.8731242989	2.6463817786	-0.0006404408
C	2.1303924832	4.1926273721	-1.7109424529
H	2.4686936758	3.9087426547	-2.7109270958
C	-0.1439750637	4.9640398882	0.7132162616
H	-1.1395416998	5.1323520763	1.1313931485
C	-0.1497757617	4.9654008583	-0.7016570811
H	-1.1487293409	5.1346996622	-1.1113157414

C	0.7882641399	4.6421312312	-1.7051226999
H	0.3394070917	4.6221765905	-2.7016368198
C	0.8023267414	4.6387995276	1.7082590982
H	0.3617050346	4.6169691960	2.7084226589
C	3.0818620936	3.8825533652	-0.7160231665
H	3.9775593154	3.4162900114	-1.1340706169
C	3.0876776116	3.8811730211	0.6988363236
H	3.9868920767	3.4143362990	1.1086404452
C	2.1445238537	4.1895217730	1.7021663830
H	2.4910345798	3.9037990899	2.6987798671
TH	1.8509916193	-2.0838575657	0.0010909452
C	2.5774227596	-3.9399179730	-1.7066223631
H	2.1634948389	-4.0955262680	-2.7063352585
C	1.8391367800	-4.6144889938	-0.7108313750
H	0.9921766097	-5.1648330521	-1.1281420590
C	1.8353338972	-4.6169844324	0.7040319579
H	0.9861764881	-5.1688772668	1.1148313027
C	2.5683159109	-3.9461151787	1.7061953239
H	2.1491033219	-4.1052474177	2.7031272630
C	3.6218595873	-3.0008467647	1.7106987730
H	3.8200769697	-2.6051613976	2.7102549572
C	4.3710801883	-2.3391793626	0.7147811602
H	5.0081726021	-1.5552123695	1.1318215305
C	4.3748222617	-2.3365847779	-0.6999954344
H	5.0143091139	-1.5512529906	-1.1107925427
C	3.6310780523	-2.9947652640	-1.7022898495
H	3.8345770470	-2.5955685759	-2.6993475103
CL	-0.6827254345	-2.0513198452	1.7134200366
CL	-1.4361860417	1.6083227432	1.7131889357
CL	-1.4362674174	1.6078476376	-1.7139820464
CL	-0.6819099380	-2.0518499337	-1.7124780949
CL	2.1000170439	0.4329990266	-1.7153463191
CL	2.0997876805	0.4342018416	1.7157324800

$3^{\dagger}$ (DFT)

$3^{\dagger}$ (BLW)

3″ (DFT)

3″ (BLW)

3″ (BLW^{cov})

3* (DFT)

3*(BLW)

$[Th_3Cl_6]^{6+}$ (DFT) *E* = -3980.3901608169 a.u.

Th	-2.36294917	-0.77986305	0.00000000
Th	0.50612804	2.43636918	0.00000000
Th	1.85690413	-1.65644912	0.00000000
Cl	-1.43388510	1.27958709	1.54811111
Cl	-1.43388510	1.27958709	-1.54811111
Cl	1.82497613	0.60207104	-1.54816011
Cl	1.82497613	0.60207104	1.54816011
Cl	-0.39109403	-1.88174314	-1.54809711
Cl	-0.39109403	-1.88174314	1.54809711

$[Th_3Cl_6]^{6+}$ (BLW) *E* = -3979.6074561910 a.u.

TH	-2.5707355360	-0.8485166118	0.0000009333
TH	0.5503446473	2.6507866018	-0.0000004598
TH	2.0181238177	-1.8032985673	0.0000001567
CL	-1.4527307770	1.2954707980	1.5604903750
CL	-1.4527296820	1.2954702079	-1.5604897346
CL	1.8500398461	0.6109493021	-1.5596895225
CL	1.8500392201	0.6109495295	1.5596884969
CL	-0.3961366094	-1.9059611932	-1.5611233356
CL	-0.3961379267	-1.9059630670	1.5611230890
